# Heat Shock Protein 70 Gene Polymorphisms and Cancer Risk: A Meta-Analysis

**DOI:** 10.1155/2014/540309

**Published:** 2014-07-20

**Authors:** Lei He, Tao Deng, He-sheng Luo

**Affiliations:** Department of Gastroenterology, Renmin Hospital of Wuhan University, Wuhan 430060, China

## Abstract

The polymorphisms in the three main heat shock protein 70 (HSP70-1, HSP70-2, and HSP70-hom) genes were identified to be associated with cancer risk. However, the results are inconsistent. We perform a meta-analysis to evaluate the association between the three HSP70 polymorphisms and cancer risk. Relevant studies were identified using PubMed, Web of Science, Chinese National Knowledge Infrastructure (CNKI), and Wanfang databases up to March 29, 2014. The cancer risk associated with the HSP70 polymorphisms was estimated for each study by odds ratios (OR) together with its 95% confidence interval (CI), respectively. Twenty case-control studies from eighteen publications were included; a significant association was observed for HSP70-2 polymorphism (dominant model: OR = 1.53, 95% CI: 1.11–2.09; recessive model: OR = 1.91, 95% CI: 1.06–3.45; AG versus AA: OR = 1.38, 95% CI: 1.03–1.84; GG versus AA: OR = 2.34, 95% CI: 1.21–4.54), while there was no significant association for HSP70-1 and HSP70-hom polymorphisms. Besides, in stratification analyses by ethnicity, cancer type, and source of control, significant association was detected for HSP70-2 polymorphism, while for HSP70-hom polymorphism, we found a significant association in hospital-based population under homozygote comparison model. This meta-analysis suggests that the HSP70-2 polymorphism rather than HSP70-hom and HSP70-1 polymorphisms was associated with the risk of cancer.

## 1. Introduction

Cancer is recognized as one of the leading causes of death in economically developed countries as well as in developing countries. According to the estimation of GLOBOCAN, approximately 12.7 million new cases and 7.6 million deaths of cancer had occurred in 2008, it has become a major public health challenge [1]. Although the mechanism of carcinogenesis is still not fully understood, it has been suggested that environmental factors, interplaying with low-penetrance susceptibility genes, may be important in the development of cancer [2, 3].

Heat shock proteins (HSPs) are evolutionarily highly conserved stress proteins expressed and induced by heat shock, infection, inflammation, ischemia hypoxia, oxidative stress, carcinogens, and so on [4, 5]. Mammalian HSPs have been classified into six families according to their molecular weight. Among them, the HSP70 family is one of the most conservative and well-known HSPs. There are three main genes (HSPA1A, HSPA1B, and HSPA1L) in the human HSP70 family and the coding proteins were defined as HSP70-1, HSP70-2, and HSP70-hom, respectively. All three genes were located in class III region of the human major histocompatibility complex (MHC) on chromosome 6. HSPA1A and HSPA1B genes encode an identical protein, the heat inducible HSP70 protein, whereas the HSPA1L gene encodes a non-heat inducible protein that shares 90% sequence identity with HSP70 protein [6]. It has been proposed that the HSP70 plays an important role in tumor development, treatment, and prognosis and has distinct immunologic mechanisms affecting tumor cells and promoting cell growth [7, 8]. In cancer cells, the expression of HSP70 is abnormally high and the protein may participate in oncogenesis and in resistance to chemotherapy [9]. Several single nucleotide polymorphisms (SNPs) have been described in these genes. The most studied regions are located at positions +1267 of HSP70-2 (rs1061581), +2437 of HSP70-hom (rs2227956), and +190 of HSP70-1 (rs1043618). These SNPs could affect HSP70 expression or function and further contribute to disease susceptibility and stress tolerance [10].

To date, several studies have investigated the association between the three HSP70 polymorphisms and risk of cancer [11–31]. However, the results remain controversial. Therefore, we conduct a meta-analysis to evaluate the association between the HSP70 polymorphisms and cancer susceptibility.

## 2. Materials and Methods

### 2.1. Search Strategy

We searched for relevant studies before March 29, 2014, by using electronic PubMed, Web of Science, Chinese National Knowledge Infrastructure (CNKI), and Wanfang databases with the following terms: “heat shock protein 70 or HSP70,” “genetic polymorphism or polymorphisms or variant or SNP,” and “cancer or carcinoma or tumor.” The search was restricted to humans and without language restrictions. Additional studies were identified by a hand search of references of original or review articles on this topic. If more than one geographic or ethnic heterogeneous group was reported in one report, each was extracted separately.

### 2.2. Inclusion Criteria and Exclusion Criteria

The inclusion criteria were as follows: (1) studies that evaluated the association between the HSP70 polymorphisms and cancer, (2) a case-control study design, and (3) studies that had detailed genotype frequency of cases and controls or could be calculated from the paper text, while major exclusion criteria were (1) case-only study, case reports, and review articles, (2) studies without the raw data of the HSP70 genotype, and (3) studies that compared the HSP70 variants in precancerous lesions.

### 2.3. Data Extraction

The following information was extracted from each eligible publication: the first author's name, year of publication, country of origin, ethnicity, cancer type, source of control, genotyping methods, number of cases and controls, and Hardy-Weinberg equilibrium (HWE) in controls (*P* value). All data were extracted by two investigators independently, using the same standard. The results were compared and disagreements were resolved by consensus.

### 2.4. Statistical Analysis

The risk of cancer associated with the HSP70 polymorphisms was estimated for each study by odds ratio (OR) and 95% confidence interval (95% CI). Four different ORs were calculated: dominant model (the combined variant homozygote and heterozygote versus the wild-type homozygote), recessive model (the variant homozygote versus the combined heterozygote and wild-type homozygote), heterozygote comparison (heterozygote versus the wild-type homozygote), and homozygote comparison (variant homozygote versus the wild-type homozygote). A *χ* ^2^-test-based *Q* statistic test was performed to assess the between-study heterogeneity [32]. We also quantified the effect of heterogeneity by *I* ^2^ test. When a significant *Q* test (*P* < 0.05) or *I* ^2^ > 50% indicated heterogeneity across studies, the random effects model was used [33] or else the fixed effects model was chosen [34]. In addition, we tested whether genotype frequencies of controls were in HWE using *χ* ^2^ test. We performed stratification analyses on ethnicity, cancer type, and source of controls. Analysis of sensitivity was performed to evaluate the stability of the results. Finally, potential publication bias was investigated using Begg' funnel plot and Egger's regression test [35, 36]. *P* < 0.05 was considered statistically significant.

All analyses were performed by the Cochrane Collaboration RevMan 5.2 and STATA package version 12.0 (Stata Corporation, College Station, Texas).

## 3. Results

### 3.1. Study Characteristics

**Figure 1:**
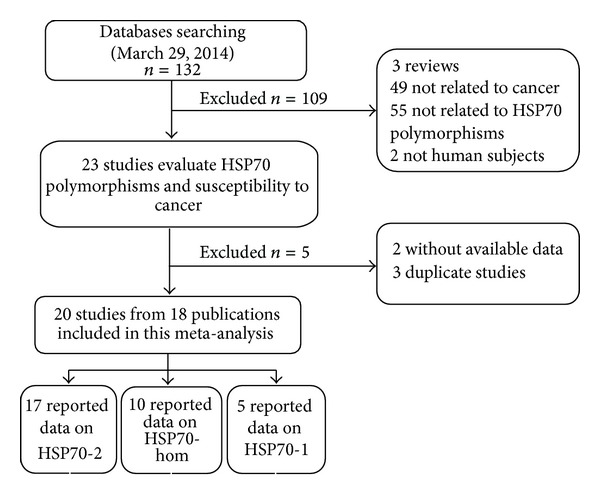
Flow chart showing study selection procedure.

After an initial search, a total of 132 published articles relevant to the topic were identified. According to the inclusion criteria, 21 studies [11–31] with full-text were included in this meta-analysis and 111 studies were excluded. The flow chart of study selection is summarized in Figure 1. As shown in Table 1, because the study by Chouchane et al. [11] included two cancer types and the study by Ucisik-Akkaya et al. [26] included two populations, we treated them separately in this meta-analysis. In addition, we excluded three studies [29–31] because they included the overlapped data with those included in the analysis [27]. Therefore, there were 17 case-control studies with 2134 cases and 2818 controls concerning HSP70-2 polymorphism, 10 studies with 2042 cases and 2661 controls concerning HSP70-hom polymorphism, and 5 studies with 1558 cases and 2075 controls concerning HSP70-1 polymorphism. Of the 19 eligible studies, four ethnicities were addressed: 8 studies on Asians, 4 studies on Europeans, 5 studies on Africans, and 3 studies on mixed populations. 3 studies focused on hepatocellular carcinoma, 3 studies on gastric cancer, 3 studies on breast cancer, and 11 studies on others (2 on lung, childhood acute lymphoblastic leukemia (ALL) and one on colorectal, pancreatic, prostate, and Kangri cancer, multiple myeloma, and non-Hodgkin's lymphoma, resp.). The distribution of genotypes in the controls was consistent with the Hardy-Weinberg equilibrium for all selected studies except for six studies [11, 19, 20, 23, 28] for HSP70-2 polymorphism and four studies [18, 20, 26] for HSP70-hom polymorphism.

### 3.2. Quantitative Data Synthesis

**Figure 2:**
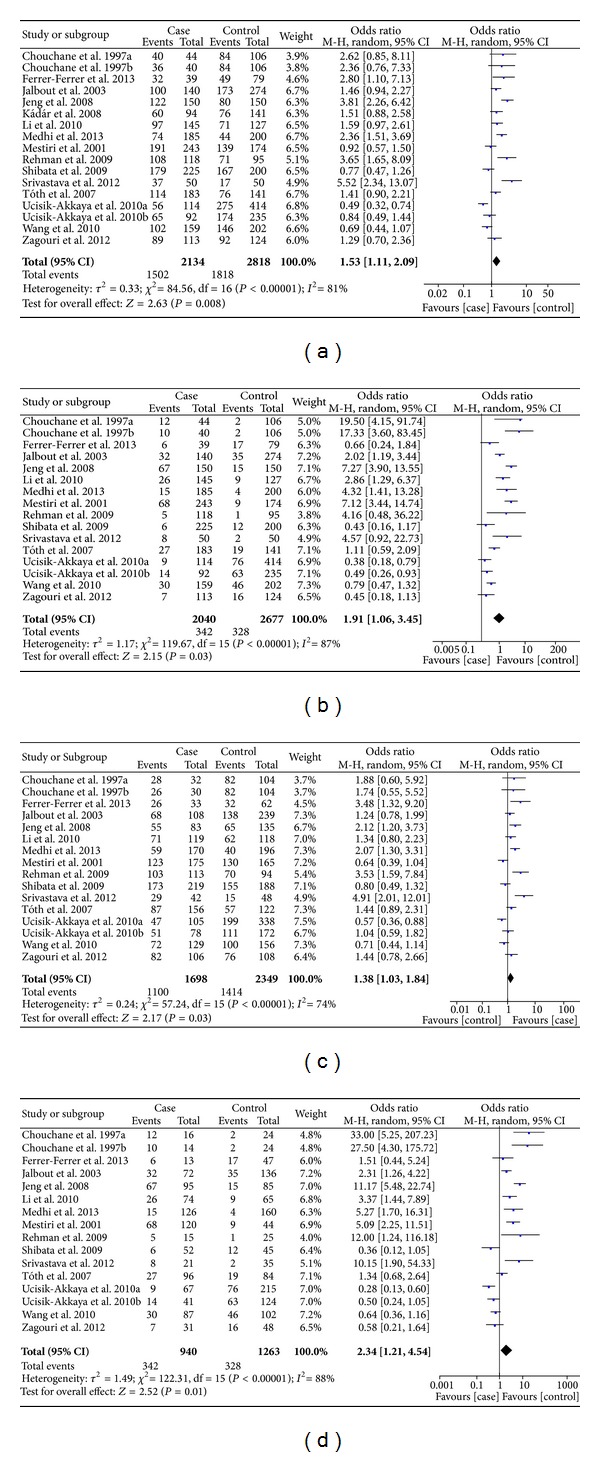
Meta-analysis of the association between HSP70-2 polymorphism and susceptibility to cancer. ((a) dominant model; (b) recessive model; (c) AG versus AA; (d) GG versus AA.)

For HSP70-2 polymorphism, 17 studies with 2134 cases and 2818 controls were identified. Overall, a significant association was found (dominant model: OR = 1.53, 95% CI: 1.11–2.09; recessive model: OR = 1.91, 95% CI: 1.06–3.45; AG versus AA: OR = 1.38, 95% CI: 1.03–1.84; GG versus AA: OR = 2.34, 95% CI: 1.21–4.54) (Figure 2). In stratified analysis by ethnicity, we found that the polymorphism was associated with an increased risk of cancer in Asians (dominant model: OR = 1.96, 95% CI: 1.10–3.51; AG versus AA: OR = 1.67, 95% CI: 1.03–2.71), and Africans (recessive model: OR = 7.06, 95% CI: 2.33–21.41; GG versus AA: OR = 7.56, 95% CI: 2.44–23.39) but not for other populations. In the stratified analysis based on cancer type, a significant association was detected in hepatocellular carcinoma (dominant model: OR = 2.41, 95% CI: 1.50–3.87; recessive model: OR = 4.98, 95% CI: 3.18–7.79; AG versus AA: OR = 1.80, 95% CI: 1.34–2.42; GG versus AA: OR = 6.07, 95% CI: 2.79–13.19), and we failed to detect any association between them among breast and other cancers. Stratification based on the source of controls showed significant associations between the polymorphism and risk of cancer in the population-based subgroup (dominant model: OR = 1.57, 95% CI: 1.06–2.34; recessive model: OR = 2.69, 95% CI: 1.21–5.97; GG versus AA: OR = 3.27, 95% CI: 1.36–7.83); however, no significant association was found in the hospital-based subgroup (Table 2).

For HSP70-hom polymorphism, 10 studies with 2042 cases and 2661 controls were identified. Overall, no significant association was found under all genetic models (dominant model: OR = 0.85, 95% CI: 0.53–1.37; recessive model: OR = 1.05, 95% CI: 0.50–2.18; TC versus TT: OR = 0.84, 95% CI: 0.55–1.29; CC versus TT: OR = 0.98, 95% CI: 0.43–2.24). When the analysis was stratified by ethnicity, similar results were observed among Asian, European, African, and mixed populations. While, in stratified analysis by source of controls, a significant association was found in the hospital-based subgroup (CC versus TT: OR = 3.66, 95% CI: 1.03–13.02) but not in the population-based subgroup (Table 2).

For HSP70-1 polymorphism, 5 studies with 1558 cases and 2075 controls were identified. The pooled results suggested that no significant association was found in overall analysis (dominant model: OR = 1.30, 95% CI: 0.75–2.24; recessive model: OR = 1.02, 95% CI: 0.52–2.00; GC versus GG: OR = 1.26, 95% CI: 0.75–2.12; CC versus GG: OR = 1.13, 95% CI: 0.52–2.45) (Table 2).

### 3.3. Heterogeneity and Sensitivity Analysis

For all three polymorphisms, substantial heterogeneities were observed among overall studies in all four genetic models (HSP70-2: dominant model: *I* ^2^ = 81%, *P* < 0.00001, recessive model: *I* ^2^ = 87%, *P* < 0.00001, AG versus AA: *I* ^2^ = 74%, *P* < 0.00001, GG versus AA: *I* ^2^ = 88%, *P* < 0.00001; HSP70-hom: dominant model: *I* ^2^ = 87%, *P* < 0.00001; recessive model: *I* ^2^ = 60%, *P* = 0.008; TC versus TT: *I* ^2^ = 83%, *P* < 0.00001; CC versus TT: *I* ^2^ = 68%, *P* = 0.001; HSP70-1: dominant model: *I* ^2^ = 89%, *P* < 0.00001; recessive model: *I* ^2^ = 79%, *P* = 0.0008; GC versus GG: *I* ^2^ = 86%, *P* < 0.0001; CC versus GG: *I* ^2^ = 83%, *P* = 0.0001) (Table 2). Therefore, we conducted stratified analysis by ethnicity, cancer type, and source of the controls to find the potential sources of heterogeneity. For HSP70-2 polymorphism, we found that heterogeneity significantly reduced or removed among Africans, mixed populations, hepatocellular, and breast cancers. However, heterogeneity still exists in Asians, Europeans, other cancers, population-based, and hospital-based populations. For HSP70-hom polymorphism, heterogeneity significantly reduced or removed in mixed populations and hospital-based populations, but it still exists among Asians, Africans, and population-based populations. Then, sensitivity analysis, after removing one study at a time, was performed to evaluate the stability of the results. When excluded the study by Partida-Rodríguez et al. or Wang et al. in HSP70-1 polymorphism, the study by Medhi et al. in HSP70-hom polymorphism, the heterogeneity was effectively decreased, which suggests that the particular study may be the source of heterogeneity. In addition, no other single study influenced the pooled OR qualitatively as indicated, suggesting that the results of this meta-analysis are credible.

### 3.4. Publication Bias

Begg's funnel plot and Egger's test were performed to assess the potential publication bias in the available literature. The shape of funnel plots did not reveal any evidence of funnel plot asymmetry (data not shown). Egger's test also showed that there was no statistical significance for the evaluation of publication bias under dominant model (HSP70-2 polymorphism: *P* = 0.039, HSP70-hom polymorphism: *P* = 0.537, and HSP70-1 polymorphism: *P* = 0.367).

## 4. Discussion

To our knowledge, this is the first meta-analysis which comprehensively assessed the associations between HSP70 polymorphisms and cancer risk. In this study, we found significant associations in the overall comparison for HSP70-2 polymorphism. Individuals with the AG/GG genotype could have an increased risk of cancer. However, we failed to detect any association for HSP70-1 and HSP70-hom polymorphisms. Moreover, in the stratified analyses by several variables, including ethnicity, cancer type, and source of the controls, significant association was detected among Asians, Africans, hepatocellular carcinoma, and population-based population for HSP70-2 polymorphism, while for HSP70-hom polymorphism, we observed a significant association in hospital-based population under homozygote comparison model.

The HSP70 family is the most important and best characterized family of stress proteins. It acts as a chaperone molecule for antigenic peptides derived from tumor cells, leading to an antitumor immune recognition by cytotoxic T lymphocytes [37–39]. In addition, HSP70 is induced in tumor cells to overcome the stressful conditions faced by the tumor, such as lack of nutrients, oxygen, or antitumor immune response contributing to their survival [40]. Recently, the three main polymorphisms in these genes have investigated the association with many cancers, such as gastric, colorectal, hepatocellular, and breast cancer. As for the HSP70-2 and HSP70-hom polymorphisms, the HSP70-2 A+1267G polymorphism is a synonymous mutation located at the coding region and is likely to affect the secondary structure of mRNA, thus affecting the stability of mRNA and protein expression, and the HSP70-hom T > C polymorphism has nonsynonymous mutations, which leads to a Met to Thr substitution at position 493 in the peptide binding domain and may affect substrate binding specificity and chaperone activity of HSP70 [6]. Some investigations demonstrated that genetic alteration of the HSP70-2 and/or HSP70-hom can modulate cancer susceptibility and that the frequency of the variant genotype was significantly higher in patients when compared with controls [11, 12, 18, 22]. However, the association of Ala variants and cancer risk was not validated by others [16, 21, 25, 27, 28]. Besides, Ucisik-Akkaya et al. [26] reported that the HSP70-2 polymorphism may be a protective factor for acute lymphoblastic leukemia in both Welsh and Mexican populations. With regard to HSP70-1, the HSP70-1 G+190C polymorphism base pair lies upstream of the translation initial site [6]. In a study from Mexico, Partida-Rodríguez et al. [21] reported that HSP70-1 C/G showed significant association with gastric cancer; similarly, Wang et al. [27] suggest that HSP70-1 G+190C may contribute to individual susceptibility to lung cancer in a Chinese Han population; however, Guo et al. [13] found that the HSP70-1 polymorphism was not associated with lung cancer risk.

In this meta-analysis, we found that individuals with AG/GG genotype had a higher risk of developing cancer under all four models in HSP70-2 polymorphism; besides, in the stratified analyses by ethnicity, cancer type, and source of control, we found that G allele carriers had a higher risk of cancer than AA genotype carriers in Asians, Africans, hepatocellular carcinoma, and population-based population. With regard to HSP70-hom and HSP70-1 polymorphisms, the genotype distribution between cancer and control was not of significant difference. The inconsistent results may be attributed to differences in genetic backgrounds, environmental factors, and other factors, such as small sample size or inadequate adjustment for confounding factors. For example, the distribution of the AA genotype is about twice as frequent in the Chinese Han population, and the frequency of the GG genotype is similar, slightly above 25%, in the Costa Rican, Mexican, and Chinese population, whereas it does not reach 10% in the Tunisian, Indian, and Japanese groups [12]. Furthermore, the interaction among some other SNPs might affect the relationship of each polymorphism included with the development of cancer. Some reports suggest possible linkage disequilibrium between HSP70 and TNF SNPs, since TNF and HSP70 gene families are located just 600 kb apart from each other [11, 19], which indicate that TNF and HSP70 may act as endogenous tumor promoters in vivo. HSP70 effects, thereby, may be modulated by the TNF genotype. Additionally, because only few studies on subgroup (such as hepatocellular carcinoma, breast cancer, Africans, and hospital-based) were included, the results should be interpreted with caution, and more studies are needed.

Two significant issues should be addressed in this study, that is, heterogeneity and publication bias, which may influence the results of meta-analysis. We do not detect a significant publication bias in this meta-analysis, suggesting the reliability of our results. With regard to heterogeneity, substantial heterogeneities were observed among overall studies in all four genetic models for all three polymorphisms, when stratified analysis by ethnicity, cancer type and source of the controls were conducted. For HSP70-2 polymorphism, we found that heterogeneity significantly reduced or removed among Africans, mixed populations, and hepatocellular and breast cancers but not among Asians, Europeans, other cancers, population-based, and hospital-based populations. For HSP70-hom polymorphism, heterogeneity significantly reduced or removed in mixed populations and hospital-based populations, but it still exists among Asians, Africans, and population-based populations. When excluded the study by Partida-Rodríguez et al. or Wang et al. in HSP70-1 polymorphism, the study by Medhi et al. in HSP70-hom polymorphism, the heterogeneity was effectively decreased. The results above suggest that different ethnicity, tumor types, control selection, and particular study may be the source of heterogeneity.

This meta-analysis has limitations that must be acknowledged. First, because of incomplete raw data or publication limitations, some relevant studies could not be included in our analysis. Second, the controls included in our analysis were selected variously either from populations or hospitals. Therefore, misclassification bias was possible because these studies may have included control groups who have different risks of developing cancer. Third, our results were based on unadjusted estimates, while lacking of the information (such as age, gender, family history and other risk factors) for the date analysis may cause serious confounding bias.

In summary, this meta-analysis suggested that the HSP70-2 polymorphism rather than HSP70-hom and HSP70-1 polymorphisms was associated with the risk of cancer. However, large and well-designed studies taking into consideration gene-gene and gene-environment interactions are warranted to validate our findings.

## Figures and Tables

**Table 1:** Characteristics of studies included in the meta-analysis.

Study	Year	Country	Ethnicity	Cancer type	Source of controls	Genotype methods	Genotype (case/control)				HWE
							Total	WT Ho	Ht	VR Ho	(*P* value)
HSP70-2								AA	AG	GG	

Chouchane et al. [11]	1997a	Tunisia	African	Non-Hodgkin's lymphoma	PB	AFLP	44/106	4/22	28/82	12/2	0
Chouchane et al. [11]	1997b	Tunisia	African	Breast	PB	AFLP	40/106	4/22	26/82	10/2	0
Ferrer-Ferrer et al. [12]	2013	Costa Rica	Mixed	Gastric	HB	RFLP	39/79	7/30	26/32	6/17	0.137
Jalbout et al. [14]	2003	Tunisia	African	Nasopharyngeal	PB	AFLP	140/274	40/101	68/138	32/35	0.251
Jeng et al. [15]	2008	Taiwan (China)	Asian	Hepatocellular	HB	AFLP	150/150	28/70	55/65	67/15	0.987
Kádár et al. [16]	2008	Hungary	European	Multiple myeloma	PB	PCR-RFLP	94/141	34/65	60/76∗		NA
Li et al. [17]	2010	China	Asian	Hepatocellular	HB	PCR	145/127	48/56	71/62	26/9	0.139
Medhi et al. [18]	2013	India	Asian	Hepatocellular	PB	PCR-RFLP	185/200	111/156	59/40	15/4	0.453
Mestiri et al. [19]	2001	Tunisia	African	Breast	PB	PCR	243/174	52/35	123/130	68/9	0
Rehman et al. [20]	2009	India	Aisian	Kangri cancer	PB	PCR-RFLP	118/95	10/24	103/70	5/1	0
Shibata et al. [23]	2009	Japan	Asian	Gastric	HB	PCR-RFLP	225/200	46/33	173/155	6/12	0
Srivastava et al. [24]	2012	India	Asian	Pancreatic	PB	PCR-RFLP	50/50	13/33	29/15	8/2	0.858
Tóth et al. [25]	2007	Hungary	European	Colorectal	PB	PCR-RFLP	183/141	69/65	87/57	27/19	0.258
Ucisik-Akkaya et al. [26]	2010a	UK	European	Childhood ALL	PB	PCR-RFLP	114/414	58/139	47/199	9/76	0.747
Ucisik-Akkaya et al. [26]	2010b	Mexico	Mixed	Childhood ALL	PB	PCR-RFLP	92/235	27/61	51/111	14/63	0.397
Wang et al. [27]	2010	China	Asian	Lung	HB	PCR-RFLP	159/202	57/56	72/100	30/46	0.915
Zagouri et al. [28]	2012	Greece	European	Breast	HB	PCR	113/124	24/32	82/76	7/16	0.006

HSP70-hom								TT	TC	CC	

Chouchane et al. [11]	1997a	Tunisia	African	Non-Hodgkin's lymphoma	PB	AFLP	44/106	31/98	10/8	3/0	0.686
Chouchane et al. [11]	1997b	Tunisia	African	Breast	PB	AFLP	40/106	31/98	8/8	1/0	0.686
Ferrer-Ferrer et al. [12]	2013	Costa Rica	Mixed	Gastric	HB	RFLP	39/79	34/55	5/23	0/1	0.409
Guo et al. [13]	2011	China	Asian	Lung	PB	TaqMan	1152/1152	674/695	412/411	66/46	0.124
Medhi et al. [18]	2013	India	Asian	Hepatocellular	PB	PCR-RFLP	185/200	178/144	7/42	0/14	0.0001
Rehman et al. [20]	2009	India	Aisian	Kangri cancer	PB	PCR-RFLP	118/95	60/28	56/60	2/7	0.001
Sfar et al. [22]	2010	Tunisia	African	Prostate	PB	PCR-RFLP	101/105	77/65	20/32	4/8	0.164
Ucisik-Akkaya et al. [26]	2010a	UK	European	Childhood ALL	PB	TaqMan	105/371	67/246	30/103	8/22	0.015
Ucisik-Akkaya et al. [26]	2010b	Mexico	Mixed	Childhood ALL	PB	TaqMan	99/245	87/214	11/27	1/4	0.008
Wang et al. [27]	2010	China	Asian	Lung	HB	PCR-RFLP	159/202	95/141	56/59	8/2	0.120

HSP70-1								GG	GC	CC	

Guo et al. [13]	2011	China	Asian	Lung	PB	TaqMan	1152/1152	589/564	457/486	106/102	0.853
Partida-Rodríguez et al. [21]	2010	Mexico	Mixed	Gastric	HB	PCR-RFLP	42/106	4/59	37/41	1/6	0.746
Ucisik-Akkaya et al. [26]	2010a	UK	European	Childhood ALL	PB	TaqMan	106/365	47/137	45/162	14/66	0.139
Ucisik-Akkaya et al. [26]	2010b	Mexico	Mixed	Childhood ALL	PB	TaqMan	99/250	62/127	32/98	5/25	0.347
Wang et al. [27]	2010	China	Asian	Lung	HB	PCR-RFLP	159/202	57/104	65/82	37/16	0.977

HWE, Hardy-Weinberg equilibrium; *P*_HWE_ was calculated by goodness-of fit *χ* ^2^-test, *P*_HWE_ < 0.05 was considered statistically significant; NA, not available. Ht, heterozygote; VR Ho, variant homozygote; WT Ho, wide-type homozygote; ∗Numbers of Ht + VR Ho.

**Table 2:** Summary of ORs of the HSP70 polymorphism and cancer risk.

Variables	*N*	Dominant model			Recessive model			Ht versus WT Ho			VR Ho versus WT Ho		
		OR (95% CI)	*P* ^a^	*I* ^2^	OR (95% CI)	*P* ^a^	*I* ^2^	OR (95% CI)	*P* ^a^	*I* ^2^	OR (95% CI)	*P* ^a^	*I* ^2^
*HSP70-2 *													
Total	17	1.53 (1.11, 2.09)	<0.00001	81	1.91 (1.06, 3.45)	<0.00001	87	1.38 (1.03, 1.84)	<0.00001	74	2.34 (1.21, 4.54)	<0.00001	88
Ethnicity													
Asian	7	1.96 (1.10, 3.51)	<0.00001	87	2.34 (0.94, 5.85)	<0.00001	86	1.67 (1.03, 2.71)	<0.0001	80	3.12 (0.99, 9.77)	<0.00001	89
European	4	1.06 (0.59, 1.89)	0.0009	82	0.59 (0.29, 1.22)	0.06	64	1.04 (0.54, 1.99)	0.007	80	0.61 (0.23, 1.66)	0.01	78
African	4	1.34 (1.00, 1.80)	0.19	37	7.06 (2.33, 21.41)	0.0009	82	1.09 (0.66, 1.82)	0.11	51	7.56 (2.44, 23.39)	0.005	77
Mixed	2	1.44 (0.45, 4.65)	0.03	79	0.53 (0.31, 0.91)	0.62	0	1.78 (0.55, 5.78)	0.03	78	0.77 (0.27, 2.23)	0.13	55
Cancer type													
Hepatocellular	3	2.41 (1.50, 3.87)	0.06	65	4.98 (3.18, 7.79)	0.19	40	1.80 (1.34, 2.42)	0.38	0	6.07 (2.79, 13.19)	0.10	57
Breast	3	1.16 (0.81, 1.64)	0.29	19	3.61 (0.43, 30.28)	<0.00001	92	1.06 (0.55, 2.05)	0.07	62	3.86 (0.56, 26.43)	0.0002	88
Others	11	1.39 (0.93, 2.07)	<0.00001	82	1.13 (0.64, 2.00)	<0.00001	78	1.37 (0.92, 2.06)	<0.00001	78	1.43 (0.70, 2.93)	<0.00001	83
Source of control													
PB	11	1.57 (1.06, 2.34)	<0.00001	81	2.69 (1.21, 5.97)	<0.00001	87	1.44 (0.95, 2.16)	<0.00001	78	3.27 (1.36, 7.83)	<0.00001	87
HB	6	1.46 (0.83, 2.57)	<0.00001	84	1.17 (0.44, 3.09)	<0.00001	90	1.31 (0.86, 2.00)	0.006	69	1.43 (0.46, 4.47)	<0.00001	90
*HSP70-hom *													
Total	10	0.85 (0.53, 1.37)	<0.00001	87	1.05 (0.50, 2.18)	0.008	60	0.84 (0.55, 1.29)	<0.00001	83	0.98 (0.43, 2.24)	0.001	68
Ethnicity													
Asian	4	0.55 (0.24, 1.28)	<0.00001	93	0.68 (0.14, 3.32)	0.001	81	0.59 (0.28, 1.23)	<0.00001	91	0.57 (0.09, 3.42)	0.0002	85
European	1	1.12 (0.71, 1.75)	NA	NA	1.31 (0.56, 3.03)	NA	NA	1.07 (0.66, 1.74)	NA	NA	1.34 (0.57, 3.13)	NA	NA
African	3	2.02 (0.41, 9.94)	<0.0001	90	3.03 (0.24, 38.65)	0.04	70	1.79 (0.44, 7.29)	0.0006	86	3.33 (0.20, 55.83)	0.02	75
Mixed	2	0.61 (0.22, 1.68)	0.11	61	0.63 (0.10, 3.88)	0.97	0	0.64 (0.23, 1.77)	0.11	61	0.59 (0.10, 3.64)	0.95	0
Source of control													
PB	8	0.86 (0.49, 1.52)	<0.00001	89	0.87 (0.39, 1.94)	0.008	63	0.85 (0.51, 1.42)	<0.00001	85	0.79 (0.32, 1.98)	0.001	71
HB	2	0.78 (0.18, 3.50)	0.008	86	3.58 (0.99, 12.93)	0.26	23	0.77 (0.20, 2.97)	0.02	82	3.66 (1.03, 13.02)	0.19	42
*HSP70-1 *													
Total	5	1.30 (0.75, 2.24)	<0.00001	89	1.02 (0.52, 2.00)	0.0008	79	1.26 (0.75, 2.12)	<0.0001	86	1.13 (0.52, 2.45)	0.0001	83

*N*: number of studies; ^a^Test for heterogeneity. CI, confidence interval; OR, odds ratio; Ht + VR Ho versus WT Ho, dominant model; VR Ho versus Ht + WT Ho, recessive model; NA, not applicable.
